# Statistical Analysis and Health Risk Assessment: Vegetables Irrigated with Wastewater in Kirri Shamozai, Pakistan

**DOI:** 10.3390/toxics11110899

**Published:** 2023-11-02

**Authors:** Mehak Nawaz Khan, Muhammad Anis Aslam, Imran Zada, Thamer H. Albekairi

**Affiliations:** 1 Shanghai Key Laboratory of Hydrogen Science & Center of Hydrogen Science, School of Materials Science and Engineering, Shanghai Jiao Tong University, Shanghai 200240, China; 2 State Key Laboratory of Metal Matrix Composites, School of Materials Science and Engineering, Shanghai Jiao Tong University, Shanghai 200240, China; 3 Department of Pharmacology and Toxicology, College of Pharmacy, King Saud University, P.O. Box 2455, Riyadh 11451, Saudi Arabia

**Keywords:** daily intake, health risk, wastewater, vegetables, heavy metals, Kirri Shamozai

## Abstract

One of the primary environmental routes through which humans are exposed to metals and may be exposed to health risks is the food chain’s contamination with heavy metals. The study observed the risks posed by contaminants in vegetables produced in soil that received wastewater irrigation, as well as their origins and the human health impacts. Eight harmful metals (Cu, Fe, Zn, Mn, Pb, Cd, Ni, and Cr) were tested for concentration levels in water, soil, and vegetable samples using analytical techniques and an atomic absorption spectrophotometer. The present study investigated the potential health implications associated with the consumption of vegetables irrigated using wastewater containing heavy metals. The results indicated a notable accumulation of heavy metals in plant and soil samples obtained from Kirri Shamozai, Pakistan. In comparison to vegetables cultivated in soil irrigated with fresh water, the concentration levels of heavy metals in vegetables grown on soil irrigated with untreated wastewater were considerably higher at (*P* ≤ 0.001) and above the World Health Organization (WHO) recommended limits. The results showed that heavy metals had significantly accumulated in the soil and had permeated into the crops. Heavy metal concentrations in vegetables cultivated on land irrigated with wastewater were more significant than those grown on land irrigated with freshwater. They exceeded US EPA and World Health Organization (WHO) limits. PCA results for Pb, Cu, and Cr are the main issues impacting water quality and health hazards. The PCA results show that the soil has an extensive loading of heavy metals Cd, Ni, and Mn.

## 1. Introduction

Kirri Shamozai is a small rural town located in the district of Dera Ismail Khan (D.I. Khan), Khyber Pakhtunkhwa (KPK) province of Pakistan. The area is known for its rich mineral deposits and mining activities, which can lead to heavy metal contamination. Heavy metals are naturally occurring elements that can have toxic effects on living organisms at high concentrations. In animals, heavy metal contamination can cause various health problems, including reproductive issues, neurological disorders, and decreased immune function. This can lead to reduced population sizes and biodiversity loss in the area. Microorganisms play a vigorous role in the ecosystem, and heavy metal contamination can significantly impact their populations. Some species of microorganisms can thrive in heavy-metal-contaminated environments, while others may be killed off. This can have implications for nutrient cycling and other ecological processes. In summary, heavy metal contamination in Kirri Shamozai can have a significant taxonomical impact, affecting plants, animals, and microorganisms in the area; monitoring and mitigating heavy metal contamination is important to protect the ecosystem and human health. Heavy metals are toxic elements such as Cu, Ni, Pb, and Cr that are released into the environment through industrial activities, mining, and agricultural practices. The taxonomic impact of heavy metals in Kirri Shamozai can be seen through their effects on various organisms. Heavy metals can accrue in the tissues of animals and plants, leading to reduced growth and reproduction, changes in behavior, and even death. This can travel along the food chain, affecting the abundance and diversity of species in the ecosystem. In plants, heavy metal toxicity can cause stunted growth, chlorosis, and reduced photosynthesis, leading to decreased yields and crop failure. Heavy metals can also alter the soil microbiota, affecting nutrient cycling and reducing soil fertility. In animals, heavy metal toxicity can affect the nervous system and cause behavioral changes and neurological damage. Heavy metals can also accumulate in the tissues of animals, including fish, which can lead to bioaccumulation at higher trophic levels, such as birds and mammals. This can lead to reduced populations and even local extinctions. For example, a study on the effects of heavy metals on soil microbial diversity found that heavy metal pollution reduced the abundance and diversity of soil bacteria and fungi [1,2,3]. Another study on the effects of heavy metals on fish found that heavy metal pollution reduced the abundance and diversity of fish species in the area. In conclusion, heavy metal pollution in Kirri Shamozai has had a significant taxonomic impact on the ecosystem. It has led to reduced growth and reproduction of plants, altered soil microbiota, reduced abundance and diversity of animal species, and even local extinctions. It is essential to mitigate heavy metal pollution in the region to protect the biodiversity and ecological health of the area. Irrigating vegetables with wastewater can lead to the accumulation of heavy metals in crops, which can pose health risks to the people who consume them. Even in small concentrations, heavy metals like lead, cadmium, arsenic, and mercury are harmful to the human body and can build up over time in the body, causing a variety of health issues. In the area of Kirri Shamozai, Pakistan, where wastewater is used for irrigation, studies have shown high levels of heavy metals in vegetables, including spinach, radishes, carrots, and cauliflower. Therefore, it is important to avoid consuming vegetables grown with wastewater and instead use fresh water for irrigation [4].

**Figure 1:**
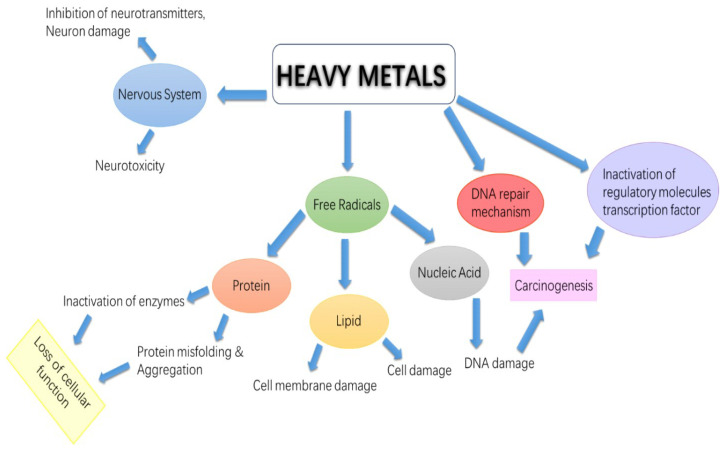
Exposure to heavy metals in humans [15].

Several factors contribute to the heavy metal poisoning of agricultural soil. These causes include the use of organic pesticides, the removal of industrial waste from cities, mining, the smelting process, air pollution from cars, and the burning of fossil fuels. One of the main contaminants in our food supply is heavy metals [5]. Metal traces have a crucial role in biological processes, especially those involving copper, manganese, lead, and chromium. Yet, necessary metals become poisonous to organisms when they reach a specific threshold [6,7,8]. As a result of fast industrial expansion, several emerging nations, including Pakistan, Bangladesh, India, Egypt, and Pakistan, are facing heavy metal poisoning in their flora and fauna. This directly affects the population’s health, the environment, and the quality of food [9]. A portion of the collected heavy metals might be absorbed by the soil, deposited on leafy surfaces, or transferred into vegetables through roots. The solubility of trace metals in soil, soil pH, and plant development characteristics are just a few examples of the many variables that affect the transmission of heavy metals through roots [10]. Additionally, crops may be more prone to contamination during the marketing and storing processes due to heavy metal deposition on the surface of crops from vehicle and industrial pollutants. As a result, the soil/vegetable system offers the best illustration possible of abiotic–biotic relationships. A key component of agricultural farming is soil, which is frequently contaminated with trace metals from both point and non-point sources. Due to a dearth of efficient purification facilities, untreated wastewater from domestic and commercial operations seeps directly into the water supply [11]. The use of trace elements in biological processes is essential to their correct functioning. When present in the organism, these trace metals trigger the activity of enzymes. All trace metals, both essential and superfluous, have toxic amounts that interfere with biological functions, damage cell membranes, and alter the three-dimensional structure of enzymes [12]. Arsenic alters the makeup of white and red blood cells in modest amounts. Arsenic in high doses causes brownish skin and black patches on the bottoms of the feet, while low doses cause serious digestive issues. Vegetables are heavily dependent on metals like Mo, Co, Fe, and Cu. When Ni concentration levels are minimal and Ni is present in abundance, the necessary activities of enzymes are activated [13]. Cleaning up surfaces and wastewater is crucial because vegetables are typically the first to absorb trace elements through soil and water. This is a challenging procedure due to several technological and societal factors. Wastewater is used by farmers in Pakistan and other emerging nations to fertilize crops. It is anticipated that this study will be helpful in establishing the amounts of chemical contamination in vegetables cultivated on land irrigated with effluent in peri-urban areas. The health danger connected to eating contaminated vegetables in a peri-urban region was identified, as well as the transfer factor of trace elements from earth to vegetables [14]. Figure 1 depicts the route of human contact with heavy metals. Vegetables with trace elements have protective benefits that are harmful to people. The current study was conducted in the Kiri Shamozai region of Dera Ismail Khan (D.I.K.), a small city in Khyber Pakhtun Khwa (KPK), Pakistan, from April 2022 to June 2023, to quantify heavy metals in wastewater and vegetables produced on wastewater-irrigated soil. Various hazard quotients were calculated to evaluate the health risks associated with the daily consumption of heavy metals from contaminated vegetables. The novelty of this research lies in the fact that Kirri Shamozai, an agricultural region characterized by its backwardness and high population density, has not been previously examined or assessed for potential health hazards. The assessment of the aforementioned region holds significant importance, as the findings unequivocally indicate that the irrigated vegetables exhibit elevated levels of heavy metals, hence posing a potential threat.

## 2. Materials and Methods

### 2.1. Study Zone

**Figure 2:**
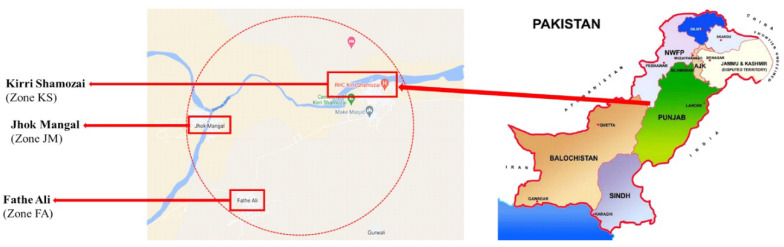
Map displaying the Kirri Shamozai study location in Dera Ismail Khan, Pakistan.

This study was executed in Kirri Shamozai, D.I. Khan, KPK, Pakistan. Farmers in Kirri Shamozai have used wastewater for irrigation since 1920. Domestic wastewater enters irrigation channels and irrigates approximately 800 acres of vegetable farms. Kirri Shamozai was divided into three zones, i.e., Kirri Shamozai denoted as “KS”, Jhok Mangal denoted as “JM”, and Fathe Ali denoted as “FA”, based on the wastewater irrigation practice and history as shown in Figure 2. Farmers in zone JM and FA move water directly from channels (Ramak Nala) into fields through a plastic pipe with installed electric machines. The selected vegetables are commonly used by the local community. To calculate physical parameters and determine the content of heavy metals, 72 wastewater samples—24 from each zone—were collected. Zone KS and zone JM irrigation water contain wastewater, domestic water, and effluents from soap and sugar mills; zone FA is watered using wastewater. For the soil sample, four sub-zones within each zone were chosen. For over forty, twenty, and fifteen years, respectively, the land in the KS, JM, and FA zones has been considered agricultural land.

### 2.2. Sample Preparation and Collection

The samples of the wastewater, soil, and vegetables were taken from various locations, including the spot where the water entered the fields from the canal [16]. The agricultural zone samples were taken from each section at ten various locations and placed in 500 mL plastic bottles. The samples were carried into the lab in an icebox and stored at 4 °C until further analysis after being treated with 5 mL concentrated HNO_3_ to prevent any bacterial heavy metal breakdown [17]. The wastewater samples were promptly transported to the laboratory. To inhibit microbial activity, the samples were treated with 1 mL of concentrated HNO_3_. The water sample was subjected to digestion by combining 50 mL of water with 10 mL of concentrated HNO_3_. The digestion process was carried out for 60 min at a temperature of 80 °C in a water bath. Subsequently, the resulting mixture was filtered using filter paper of type (Whatman No. 42). The filtrate was diluted to a final volume of 50 mL by adding deionized water.

After removing any organic matter and non-soil components like gravel, stones, wooden pieces, etc., the soil samples were collected from 30 centimeters below the surface. Polythene bags containing the earth samples were used to convey them to the lab for analysis. After chilling, the soil samples were pulverized in an electric grinder, using a 2 mm sieve to filter, and kept in clean plastic bags for characterization. The soil samples were dried at 105 °C overnight. Fresh vegetables were randomly selected from various Kirri Shamozai neighborhoods. The samples underwent testing in the lab and were washed with tap water.

The vegetables’ edible portions were diced and dehydrated in a room-temperature oven. Then, using a porcelain mortar and pestle, they were broken into discrete pieces and stored in an airtight plastic bag. The powder of a vegetable sample was likewise broken down using a hot plate and a tri-acidic solution at a 5:1:1 solution ratio. The digested samples were filtered into a 20 mL measurement flask using Whatman 42 filter paper and maintained at 5 °C under the observation of an atomic absorption spectrophotometer (Shimadzu Atomic Absorption Spectrophotometer AA-7000 Series) [18].

Eight different vegetables, including spinach (Spinacia oleracea), cabbage (Brassica oleracea), cauliflower (Brassica oleracea var. botrytis), radish (Raphanus sativus), turnip (Brassica Rapa), tinda (Praecitrullus fistulosus), carrot (Daucus carota), and lettuce (lactuca sativa) were chosen, harvested from local fields, and packed into polyethene bags. To remove any traces of dirt that could be seen, vegetable sample preparations were carefully washed with deionized water [19]. The samples were cut into pieces using a knife after the excess water was scraped from the surface of the vegetables. The samples were then dried for two days in an oven at 80 °C. Using a pestle and grinder, the dry components were ground.

### 2.3. Heavy Metal Analysis

A questionnaire was used to collect data on people’s weight, family size, age, and vegetable intake and source to conduct a nutritional assessment and assess the risk of eating vegetables grown using wastewater as irrigation. A total of 150 healthy people from the Kirri Shamozai area of Khyber Pakhtunkhwa, Pakistan, were chosen at random. The dietary questionnaire included three vegetable products, with intake calculated using the one-week recall method in kilograms per person per day. On the amended frequency of intake for a range of vegetables, information was obtained. Heavy metal concentrations (Cu, Fe, Zn, Mn, Pb, Cd, Ni, and Cr) were measured by an atomic absorption spectrophotometer.

### 2.4. Quality Control

Highest-quality chemicals were employed in this experiment. The solutions were prepared using deionized water obtained from Pakistan’s water research facility and analytical lab, Pakistan Council of Research in Water Resources (PCRWR), D.I. Khan, KPK. For quality control, each sample was examined in duplicate, and after each batch of samples, blanks and standards were used.

### 2.5. Data Analyses

#### 2.5.1. Heavy Metal Transfer Factor (HMTF)

Heavy metals must undergo a laborious procedure to pass from the soil to vegetables. The ratio of trace elements that travel to vegetables to those that are found in the soil was used to compute the transfer factor. The following algorithm was used to calculate the HMTF [20]:

HMTF = C vegetables/C soil

Metals in plants are denoted by C vegetables, whereas those in soil are denoted by C soil; HMTF stands for the transfer factor of heavy metals.

#### 2.5.2. Health Risk Assessment

The health risk percentage was used to determine the potential health risks of consuming plants contaminated with metals [21]. There is a relationship between reference dosages and computed doses. If the ratio is less than 1, it will signal that the population is on the safe side. However, if the population is at risk, HI ≥ 1.

HQ = [W plants] × [M plants]/Rf D × B [22]
[W plants] = severe (mg/L) dry plant contamination influence;
[M plants] = vegetables’ ability to absorb metals (mg/kg);
Rf D = diet containing a specified number of metals (mg/L), where B is the average body weight (Kg).

#### 2.5.3. Daily Dietary Index (DDI)

As heavy metals have the potential to damage plants, the US EPA has indicated that daily vegetable nutrition needs may be met. The following equation is utilized for the Daily Dietary Index (DDI) [23].

DDI = L × M × N/B

where L = presence of trace elements in plants, M = weight of dried vegetables, N = expected DDI of vegetables, and B = mean body mass of consumers

#### 2.5.4. Daily Intake of Metals (DIM)

DIM is used to determine the concentration of trace elements in people. We used the following equation to determine DIM.

DIM = C_feature_× C_metal_ × D_food ingestion_/B_average mass_

DIM is equal to D food consumption, D metals, C characteristics, and B average mass, where B is the average weight, C is the ratio between the values, D is the content of trace elements in vegetables, and F is the daily consumption of plants [24].

#### 2.5.5. Health Risk Index (HRI)

The health risk index (HRI) was calculated using this the following formula [25]:

HRI = DIM/Rf D

If the HRI is less than one, then there is no threat to the exposed population.

## 3. Results

### 3.1. Physicochemical Analysis and Heavy Metal Concentration Levels in Wastewater

Table S1 in the supplementary information displays the physicochemical characteristics of wastewater samples randomly selected from the three zones. The pH of wastewater had an average value of 7.5 (4 to 8.83, 7.48 to 8.39, and 7.02 to 7.98). The pH of wastewater was substantially higher than that of fresh water. The wastewater’s electrical conductivity (EC) ranged between 1695 and 1890, 1692 and 1866, and 1470 and 1372 µS cm^−1^. The EC of wastewater was above the permissible limit (1400 Scm^−1^). The total dissolved solids of wastewater ranged were 1089 to 1278, 10, and 89 to 1278, and irrigation was 606 to 782 mg/L. Total dissolved solid contents in wastewater exceeded the permissible limit (1000 mg/L). Due to carbonate and sulphate contamination, the pH of wastewater is high, because wastewater has more carbonates and sulphates compared to fresh water. As a consequence, we found that the EC and TDS of effluent water are inappropriate for agriculture, in contrast to prior studies that looked at the wastewater and soil in the Kirri Shamozai region and discovered that the EC and pH were within normal limits [26].

Trace elements Cu, Fe, Zn, Mn, Pb, Cd, Ni, and Cr in wastewater ranged from 0.28 to 0.4, 8.98 to 11.9, 0.1 to 0.34, 0.27 to 0.35, 0.2 to 0.47, 0.57 to 0.6, 0.29 to 0.39, 0.59 to 0.86, 0.25 to 0.38, 7.83 to 9.84, 0.2 to 0.38, 0.29 to 0.37, 0.2 to 0.35, 0.48 to 0.59, 0.27 to 0.59, 0.72 to 0.88, 0.15 to 0.35, 4.98 to 7.07, 0.01 to 0.09, 0.19 to 0.27, 0.05 to 0.09, 0.01 to 0.03, 0.07 to 0.2, and 0.09 to 0.22 (mg/L), correspondingly. In untreated wastewater, all toxic metals were above permissible levels. Heavy metal concentrations, in decreasing order, for the three wastewater zones were as follows: Mn > Cr > Fe > Cu > Pb > Zn > Ni > Cd [27]. The heavy metals in the wastewater (JM+FA) differed non-significantly at *P* ≤ 0.05, according to an ANOVA analysis, as shown in Table S2. A significant difference was observed when the mean concentration of groundwater of zone KS was compared to wastewater zones JM and FA.

### 3.2. Physicochemical Analysis and Heavy Metal Concentration Levels in Wastewater-Irrigated Soil

The pH of wastewater-irrigated soil ranged from 8.62 to −8.99, 8.29 to 8.65, and 7.82 to 8.20. The pH of wastewater-irrigated soil was higher than that of fresh water; this may be due to the incorporation of carbonates and sulphates from household activities involving the use of detergents. The EC ranged from 458 to 563, 477 to 570, and 308 to 450 cm^−1^ scm^−1^ for wastewater-irrigated soil. Due to its high pH and EC, the wastewater-irrigated region was not suitable for cultivation. Organic matter (OM) in wastewater-irrigated soil ranged from 1.05 to 2.59, 1.09 to 279, and 1.40 to 3.09 [28]. The pH and EC of soil samples collected were found to be similar in the locations of Kirri Shamozai, Dera Ismail Khan, KPK, Pakistan. Researchers investigated the soil, and their findings are related to the current findings. The amount of organic matter in the soil of the Bannu district and other conclusions are comparable to those of the current study [29]. Trace elements Fe, Zn, Mn, Pb, Cd, Ni, and Cr in wastewater-irrigated soil ranged from 4.6 to 39.7, 42.9 to 46.9, 28.1 to 34.1, 289.1 to 399.7, 68 to 94, 1.7 to 3.1, 60.8 to 95.9, 73 to 121, 26.1 to 36.9, 422 to 479, 22.6 to 36.8, 270 to 350.5, 68 to 86, 1.2 to 2.8, 56.1 to 79.3, 69 to 99, 17.9 to 21.8, 178.7 to 225.6, 16.4 to 22.9, 111.3 to 126.6, 32.03 to 47.3, 1.4 to 1.9, 39.146, and 33.5 to 35.9 (mg kg^−1^), respectively. It has been noted that soil pollution caused by wastewater irrigation [30] has resulted in three heavy metals exceeding the permissible limits. The results of earlier studies were in contrast with the current research. Except for lead and manganese, the harmful elements in wastewater used to irrigate farmland were below the permissible limits, as stated in (Table S3). According to an ANOVA analysis, Ni, Mn, and Cr differed noticeably at *P* > 0.001, Pb (lead) and Cu (copper) were significantly different at *P* > 0.01, and Cu, Mn, Pb, and Cr were drastically different at *P* > 0.001 > 0.05 in soil that has been irrigated by wastewater (Table S2). The soil contamination in this study is a result of irrigated wastewater.

### 3.3. Heavy Metal Contamination of Vegetables

**Figure 3:**
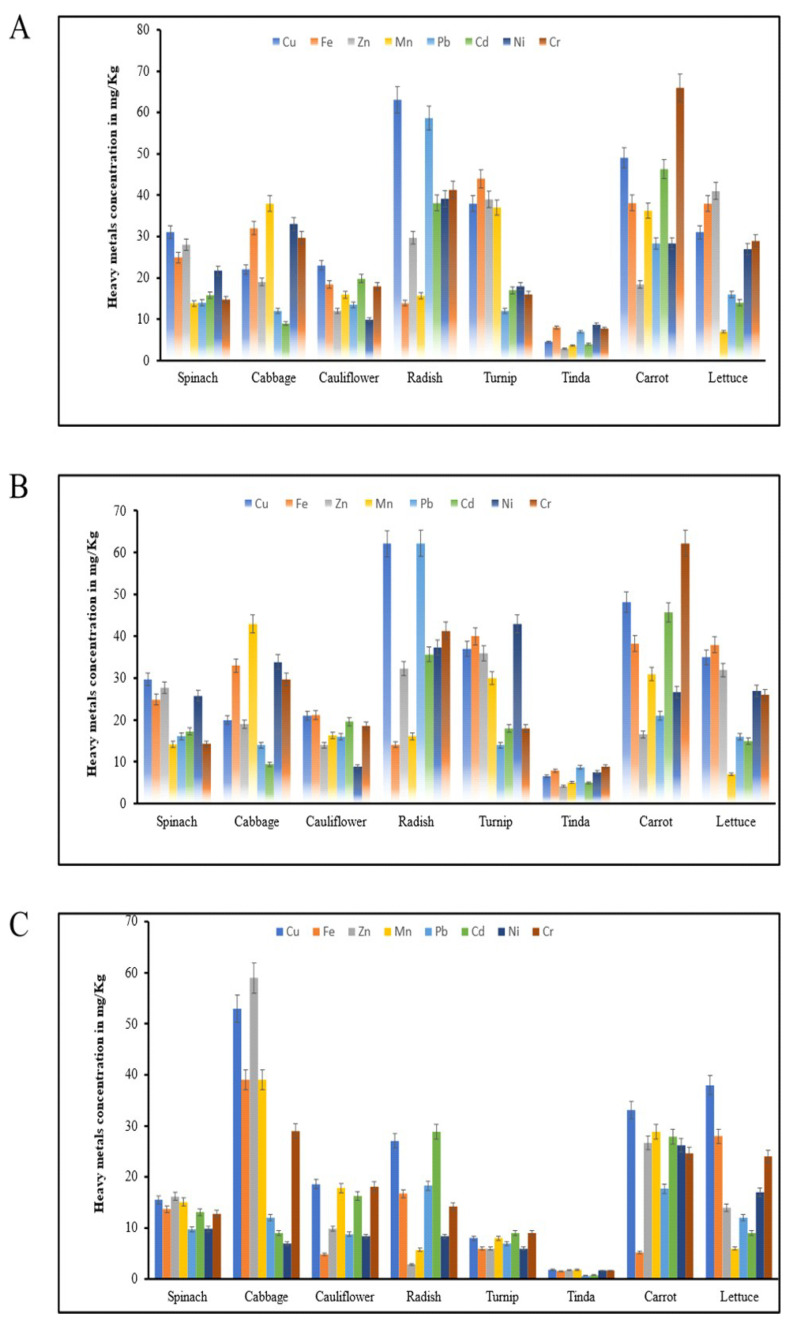
Heavy metal contents in vegetables in three zones: (**A**) KS, (**B**) FA, and (**C**) JM.

Heavy metal analysis was carried out on vegetable samples collected from three different zones. It was noted that different metals in the zones of KS, FA, and JM were above the permissible limits (Figure 3). The concentration of Cu, Fe, Zn, Mn, Pb, Cd, Ni, and Cr in zones KS, FA, and JM in eight vegetables ranged from 13.8 to 31.1, 9.00 to 38.0, 9.90 to 23.0, 13.9 to 63.1, 10.0 to 44.0, 2.8 to 10.0, 18.4 to 66.0, 7.0 to 41.0, 14.2 to 27.7, 9.4 to 43.0, 8.9 to 19.6, 16.1 to 62.2, 10.0 to 43.0, 4.20 to 11.0, 16.6 to 62.2, 7.0 to 32.0, 9.70 to 16.2, 7.0 to 59.0, 4.90 to 18.6, 2.90 to 28.9, 5.0 to 9.0, 0.70 to 1.90, 5.28 to 33.1, and 6.0 to 38.0 mg/kg, correspondingly, as shown in Table S3. The order of relative abundance was calculated for zones KS and FA: Ni > Mn > Pb > Fe > Cr > Cu > Zn > Cd. Vegetables Raphanus sativus, Brassica oleracea var. capitates, Daucus carota subsp, Spinacia oleracea, and Brassica oleracea var. botrytis had greater concentrations of the three heavy metals Cr, Cd, and Pb. The concentration of Ni was higher in Benincasa fistulosa and Spinacia oleracea. Heavy metal concentration levels were found to be higher in all vegetables of zones (KS, FA, and JM) as compared to their concentration in vegetables of zone JM. Three heavy metals, Pb, Ni, and Cd, had concentrations exceeding the WHO-permitted limit. Wastewater-irrigated vegetables have been shown to acquire higher concentrations of heavy metals, posing a significant health risk to consumers.

### 3.4. Heavy Metal Transfer Factor

When growing vegetables in soil irrigated by wastewater, HMTF is determined to estimate the health risk [31]. Table S4 lists the HMTF values for eight vegetables across three zones. In zone KS, HMTF ranged from 0.44 to 0.98, 0.02 to 0.08, 0.34 to 0.79, 0.04 to 0.19, 0.13 to 0.56, 1.22 to 4.35, 0.28 to 1.00, and 0.08 to 0.48. The HMTF values for zone FA for copper (Cu), iron (Fe), zinc (Zn), manganese (Mn), lead (Pb), cadmium (Cd), nickel (Ni), and chromium (Cr) were 0.46 to 0.97, 0.02 to 0.10, 0.33 to 0.79, 0.05 to 0.21, 0.13 to 0.57, 2.00 to 5.24, 0.26 to 0.98, and 0.08 to 0.46. In zone JM, the values of HMTF were 0.55 to 0.91, 0.03 to 0.29, 0.26 to 0.98, 0.03 to 0.25, 0.13 to 0.23, 0.49 to 1.33, 0.12 to 0.78, and 0.18 to 1.11. The results of the HMTF analysis indicate that heavy metals were deposited in zones KS, FA, and JM in the following order: Cd > Cu > Zn > Pb > Ni > Mn > Cr > Fe.

### 3.5. DIM and HRI for Vegetables

The daily heavy metal intake was calculated according to the number of vegetables consumed. After ingesting food crops produced in wastewater-irrigated soil, significantly higher DIM values for heavy metals were reported. The DIM from eating vegetables is shown in Tables S5–S7 for adults and children. The DIM (daily intake of metals) in wastewater-irrigated vegetables (zone KS) of Cu, Fe, Zn, Mn, Pb, Cd, Ni, and Cr ranged from 7.23 × 10^−3^ to 1.63× 10^−2^, 4.71× 10^−3^ to 1.99× 10^−2^, 5.18× 10^−3^ to 1.20× 10^−2^, 7.28 × 10^−3^ to 3.30 × 10^−2^, 5.24 × 10^−3^ to 2.30 × 10^−2^, 1.47 × 10^−3^ to 5.24 × 10^−3^, 9.64 × 10^−3^ to 3.46 × 10^−2^, and 3.67 × 10^−3^ to 2.15 × 10^−2^, respectively, for adults. For children, it ranged from 8.32 × 10^−3^ to 1.88 × 10^−2^, 8.32 × 10^−3^ to 1.88 × 10^−2^, 5.97 × 10^−3^ to 1.39 × 10^−2^, 8.38 × 10^3^ to 3.81 × 10^−2^, 6.03 × 10^−3^ to 2.65 × 10^−2^, 1.69 × 10^−3^ to 6.03 × 10^−3^, 1.11 × 10^−2^ to 3.98 × 10^−2^, and 4.22 × 10^−3^ to 2.47 × 10^−2^, respectively (Table S6). The DIM (daily intake of metals) of Cu, Fe, Zn, Mn, Pb, Cd, Ni, and Cr in zone FA ranged from 7.44 × 10^3^ to 1.56 × 10^−2^, 4.92 × 10^−3^ to 2.25 × 10^−2^, 4.66 × 10^−3^ to 1.11 × 10^−2^, 7.38 × 10^−3^ to 3.26 × 10^−2^, 5.24 × 10^−3^ to 2.25 × 10^−2^, 2.20 × 10^−3^ to 5.76 × 10^−3^, 8.69 × 10^−3^ to 3.26 × 10^−2^, and 3.67 × 10^−3^ to 1.99 × 10^−2^, respectively, for adults, while for children it was in the range of 8.56 × 10^−3^ to 1.79 × 10^−2^, 5.67 × 10^−3^ to 2.59 × 10^−2^, 5.37 × 10^−3^ to 1.28 × 10^−2^, 8.50 × 10^−3^ to 3.75 × 10^−2^, 6.03 × 10^−3^ to 2.59 × 10^−2^, 2.53 × 10^−3^ to 6.63 × 10^−3^, 1.00 × 10^−2^ to 3.75 × 10^−2^, and 4.22 × 10^−3^ to 2.29 × 10^−2^, respectively (Table S6 ). The daily intake of metals Cu, Fe, Zn, Mn, Pb, Cd, Ni, and Cr in zone JM ranged from 5.08 × 10^−3^ to 8.48 × 10^−3^, 3.67 × 10^−3^ to 3.09 × 10^−2^, 2.57 × 10^−3^ to 9.74 × 10^−3^, 1.52 × 10^−3^ to 1.51 × 10^−2^, 2.62 × 10^−3^ to 4.71 × 10^−3^, 3.67 × 10^−4^ to 9.95 × 10^−4^, 2.76 × 10^−3^ to 1.73 × 10^−2^, and 3.14 × 10^−3^ to 1.99 × 10^−2^ for adults, while for children it ranged from 5.85 × 10^−3^ to 9.77 × 10^−3^, 4.22 × 10^−3^ to 3.56 × 10^−2^, 2.95 × 10^−3^ to 1.12 × 10^−2^, 1.75 × 10^−3^ to 1.74 × 10^−2^, 3.02 × 10^−3^ to 5.43 × 10^−3^, 4.22 × 10^−4^ to 1.15 × 10^−3^, 3.18 × 10^−3^ to 2.00 × 10^−2^, and 3.62 × 10^−3^ to 2.29 × 10^−2^, respectively (Table S7). In wastewater-irrigated vegetables, the DIM for adults as well as children was higher than the appropriate daily intake rates for Pb, Cd, and Ni, but it was lower than the permissible daily intake rates for all other metals. Eating common vegetables grown in wastewater-irrigated regions did not cause harm as DIM levels were below the US EPA and IRIS limits. The consequences of the present study for the most harmful elements to human health—lead, cadmium, and nickel—were similar to previous findings [32].

The health risk index for vegetable consumption for both adults and children is shown in Tables S5–S7. The HRI in wastewater-irrigated vegetables (zone-KS) for Cu, Fe, Zn, Mn, Pb, Cd, Ni, and Cr ranged from 1.81 × 10^−1^ to 4.07 × 10^−1^, 6.73 × 10^−3^ to 2.84 × 10^−2^, 1.73 × 10^−2^ to 4.01 × 10^−2^, 2.21 × 10^−1^ to 1.00, 1.31 to 5.76, 1.47 to 5.24, 4.82 × 10^−1^ to 1.73, and 2.44 × 10^−3^ to 1.43 × 10^−2^. It ranged for adults and children from 2.08 × 10^−1^ to 4.69 × 10^−1^, 7.75 × 10^−3^ to 3.27 × 10^−2^, 1.99 × 10^−2^ to 4.62 × 10^−2^, 2.54 × 10^−1^ to 1.15, 1.51 to 6.63, 1.69 to 6.03, 5.55 × 10^−1^ to 1.99, and 2.81 × 10^−3^ to 1.65 × 10^−2^, respectively (Table S5). The HRI in zone FA vegetables for Cu, Fe, Zn, Mn, Pb, Cd, Ni, and Cr ranged from 1.86 × 10^−1^ to 3.89 × 10^−1^, 7.03 × 10^−3^ to 3.22 × 10^−2^, 1.55 × 10^−2^ to 3.70 × 10^−2^, 2.24 × 10^−1^ to 9.87 × 10^−1^, 1.31 to 5.63, 2.20 to 5.76, 4.35 × 10^−1^ to 1.63, and 2.44 × 10^−3^ to 1.33 × 10^−2^. It ranged, for adults and children, from 2.14 × 10^−1^ to 4.48 × 10^−1^, 8.10 × 10^−3^ to 3.70 × 10^−2^, 1.79 × 10^−2^ to 4.26 × 10^−2^, 2.58 × 10^−1^ to 1.14, 1.51 to 6.48, 2.53 to 6.63, 5.01 × 10^−1^ to 1.88, and 2.81 × 10^−3^ to 1.53 ×10^−2^, respectively (Table S6). The HRI (health risk index) in zone JM vegetables for Cu, Fe, Zn, Mn, Pb, Cd, Ni, and Cr ranged from 1.27 × 10^−1^ to 2.12 × 10^−1^, 5.24 × 10^−3^ to 4.41 × 10^−2^, 8.55 × 10^−3^ to 3.25 × 10^−2^, 4.60 × 10^−2^ to 4.59 × 10^−1^, 6.55 × 10^−1^ to 1.18, 3.67 × 10^−1^ to 9.95 × 10^−1^, 1.38 × 10^−1^ to 8.67 × 10^−1^, and 2.09 × 10^−3^ to 1.33 × 10^−2^ for adults and children ranged from 1.46 × 10^−1^ to 2.44 × 10^−1^, 6.03 × 10^−3^ to 5.08 × 10^−2^, 9.85 × 10^−3^ to 3.74 × 10^−2^, 5.30 × 10^−2^ to 5.28 × 10^−1^, 7.54 × 10^−1^ to 1.36, 4.22 × 10^−1^ to 1.15, 1.59 × 10^−1^ to 9.98 × 10^−1^, and 2.41 × 10^−3^ to 1.53 × 10^−2^, respectively (Table S7). Tables S5–S7 show the average amount of heavy metals consumed through vegetables. Table S5 displays the metal intake for both adults and children (Cu, Fe, Zn, Mn, Pb, Cd, Ni, and Cr). Except for Mn, Pb, Cd, and Ni, which were discovered in excess for adults in Spinacia oleracea and Brassica oleracea, the HRI study revealed that the metal intake in zone KS was within the WHO-permitted level. Some metals, such as Pb, Cd, and Ni, were also detected in excess for children. Vegetables cultivated in zone KS were discovered to have heavy metals below the limit that the US EPA considers to be non-hazardous to human health. By eating tainted vegetables, children and adults were exposed to heavy metals Cd, Ni, and Pb in amounts over the legal level, endangering their health. Lactuca sativa, Spinacia oleracea, and Benincasa fistulosa all had an HRI for Cd and Pb in wastewater-irrigated plants that were >1; children’s and adults’ health may be harmed by this. The HRI (health risk index) for all heavy metals in wastewater-irrigated vegetables was 1, indicating no concerns for either adults’ or children’s health [33]. Analyzing health hazards along the food chain is crucial in Pakistan since agricultural water usage is uncontrolled. Many metals had HRI values higher than 1. This presents a risk to people even at very small concentrations. The results for Cd, Ni, and Pb, which are largely accountable for endangering human health, were in line with earlier findings. Researchers have previously shown that vegetables cultivated in wastewater can pose significant health dangers [34].

### 3.6. Cluster Analysis (CA)

**Figure 4:**
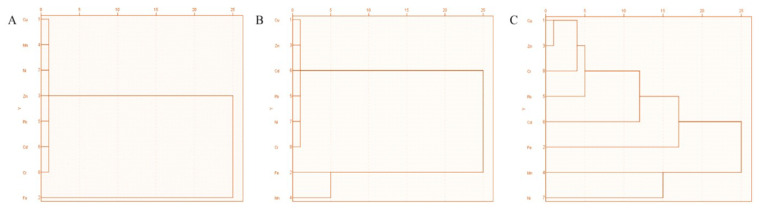
Cluster analysis of zones KS, FA, and JM: (**A**) cluster analysis of wastewater; (**B**) wastewater-irrigated soil; (**C**) wastewater-irrigated vegetables.

Cluster analysis of heavy metals is divided into three groups. The results of the cluster analysis investigation for wastewater and wastewater-irrigated soil and vegetables are shown in Figure 4 for the elements Cu, Ni, Mn, Pb, Zn, Cd, and Cr. Following the classification of the wastewater as well as wastewater-irrigated soil and vegetable samples by CA of the physicochemical properties and trace metal content, a dendrogram was formed, as shown in Figure 4. Cluster analysis revealed eight significant heavy metal clusters in the research area’s wastewater-irrigated crops. Each of the four primary clusters of heavy metals including 50% of the samples under investigation was identified. The first cluster, which comprises low-slung quality water samples, was divided into categories like “excellent water” and “unsuitable for drinking”. The sample wastewater sources are the ones that have been subjected to the most anthropogenic contamination. It has been shown that anthropogenic activities such as land use changes, geological changes, and variations in depth variables cause the pollution of water systems in the investigated region. These samples are subject to surface contamination processes from polluted springs and rivers because of the high levels of plain pollution.

### 3.7. Principal Component Analysis (PCA)

**Figure 5:**
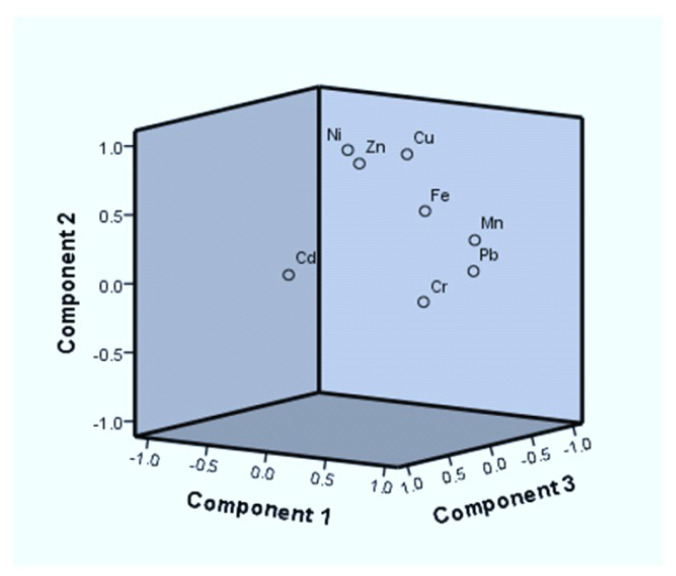
PCA of wastewater-irrigated soil of zones KS, FA, and JM.

**Figure 6:**
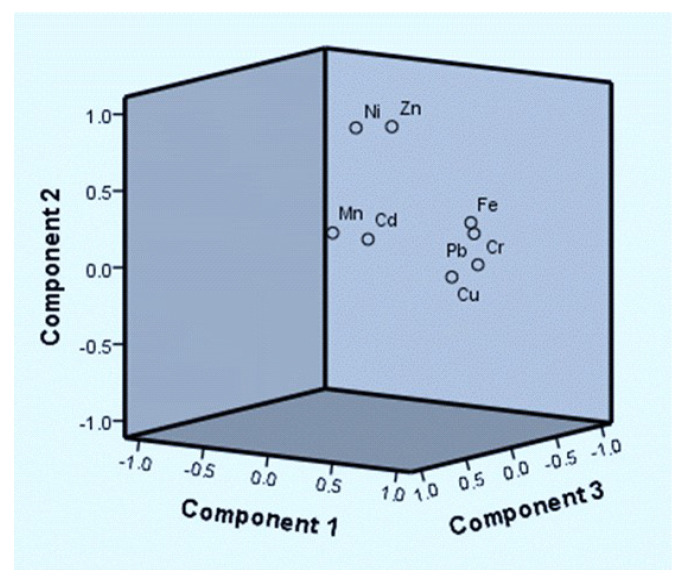
PCA of wastewater-irrigated vegetables of zones KS, FA, and JM.

The results of the principal component analysis (PCA) for samples of wastewater-irrigated soil and wastewater-irrigated vegetables are presented in Table 1 and Table 2. To classify the source of trace metals in the wastewater-irrigated soil and vegetable samples that were analyzed, the PCA and CA were combined. The significance threshold for the factor loadings is 0.05. Three unique variables of soil in heavy metals and four distinct factors of wastewater were discovered in the current study. Heavy metals accounted for 94.54% of the total. Principal component analysis shows that there is a significant loading of Cu, Ni, Mn, Pb, Cd, Cu, and Cr in wastewater, with a variability of 32.21%. The variable quantity in the correlation matrix with the highest correlations is included in this class of factors. PCA shows that the main causes of pollution in the studied areas are human activities, such as industrial, mechanical, and waste-discarding activities. According to PCA results (Figure 5 and Figure 6), Pb, Cu, and Cr are the main metals impacting water quality and health. PCA of the soil shows a large loading of heavy metals Cd, Ni, and Mn, which is responsible for 41.43% of the variability.

## 4. Discussion

The heavy metals in the wastewater (JM+FA) differ non-significantly at *P* ≤ 0.05, according to ANOVA analysis. A significant difference was observed when the mean concentration of wastewater of zone KS was compared to wastewater zones (JM+FA). Except for lead and manganese, all harmful elements in wastewater used to irrigate land were below permissible limits. According to an ANOVA analysis, Ni, Mn and Cr differed noticeably at *P* > 0.001, Cu was significantly different at *P* >0.01, and Cu, Mn, Pb, and Cr were drastically different at *P* > 0.001 > 0.05 in soil that was irrigated by wastewater. The soil contamination in this study is a result of wastewater irrigation. By eating tainted vegetables, children and adults were exposed to heavy metals Cd, Ni, and Pb in amounts over the legal level, endangering their health. Lactuca sativa, Spinacia oleracea, and Benincasa fistulosa all had an HRI for Cd and Pb in wastewater-irrigated plants that were >1; children’s and adults’ health may be harmed by this. The HRI for all heavy metals in wastewater-irrigated vegetables was 1, indicating no concerns for adult’s or children’s health. Many metals had HRI values higher than 1. Cluster analysis revealed eight significant heavy metal clusters in the research area’s wastewater-irrigated crops. Each of the four primary clusters of heavy metals included 50% of the samples under investigation. The significance threshold for the factor loadings is 0.05. Three unique variables of soil in heavy metals and four distinct factors of wastewater were discovered in the current study. Heavy metals accounted for 94.54% of the total. Principal component analysis showed that there was a significant loading of Cu, Ni, Mn, Pb, Cd, Cu, and Cr in wastewater, with a variability of 32.21%. PCA results for Pb, Cu, and Cr were the main issues impacting water quality and health hazards. PCA for the soil showed a large loading of heavy metals Cd, Ni, and Mn.

## 5. Conclusions

This study found that edible leafy vegetable sections can accumulate higher quantities of heavy metals as compared to root and fruit vegetables. Some vegetables from the study area were harmful for consumption by humans since their HRI was more than one. Therefore, further in-depth investigation is required to evaluate the problem and the risks involved. Due to ongoing wastewater irrigation practices, heavy metals accumulated to large degrees; nevertheless, the accumulation and the pace at which metals transferred from the soil to edible portions of plants varied dramatically depending on the metal. All of the studied vegetables had heavy metal concentrations below the permitted range for both the national and the international levels, except Pb and Ni. The concentration of heavy metals shows that Pb, Cd, and Ni may have a large presence in wastewater-irrigated vegetables and thus are harmful to human health when these contaminated foods are ingested. Leafy vegetables had greater levels of intake of heavy metals such as Ni, Cu, Mn, Cr, and Pb, whereas other vegetables had higher levels of Cd and Cr. In leafy vegetables, the HMTF was often higher for Pb and Ni, as seen in the following order: Pb > Cr > Ni > Mn > Cd > Fe > Cr. The mobility rate of trace metals exhibited variance in the HTMF. For determining the daily absorption of metals via the consumption of vegetables and the associated health hazards, procedures like DIM and HRI are particularly helpful. The outcomes from this study will be suitable for regulation, early execution of safety precautions, and ongoing monitoring of heavy metal discharges into soil and water. Consuming vegetables produced in Kirri Shamozai poses a considerable danger for customers. The future outlook of the research is to conduct an in-depth study and propose how to secure vegetables while minimizing health risks.

## Figures and Tables

**Table 1:** PCA for wastewater-irrigated soil of zones KS, FA, and JM in Kirri Shamozai.

Elements	Communality	Eigen	Total Variance	Cumulative TV	Factor 1	Factor 2	Factor 3
Cu	0.902	4.463	55.784	37.998	0.744	0.437	0.397
Fe	0.960	1.721	21.517	75.479	0.974	0.097	0.053
Zn	0.890	1.198	14.980	92.281	0.799	0.495	0.085
Mn	0.899	0.339			0.806	0.387	0.317
Pb	0.957	0.179			0.769	0.586	0.149
Cd	0.977	0.080			0.380	0.086	0.908
Ni	0.965	0.018			0.739	0.646	0.029
Cr	0.832	0.003			0.627	0.598	0.286

**Table 2:** PCA for wastewater-irrigated vegetables of zones KS, FA, and JM in the area of Kirri Shamozai.

Elements	Communality	Eigen	Total Variance	Cumulative TV	Factor 1	Factor 2	Factor 3
Cu	0.850	3.147	39.340	30.024	0.784	0.313	0.313
Fe	0.724	1.503	18.792	53.137	0.073	0.666	0.666
Zn	0.820	1.315	16.442	74.574	0.597	0.175	0.175
Mn	0.748	0.703			0.671	0.483	0.483
Pb	0.711	0.523			0.721	0.437	0.437
Cd	0.588	o.397			0.687	0.248	0.248
Ni	0.845	0.222			0.588	0.408	0.408
Cr	0.680	0.189			0.619	0.528	0.528

## Data Availability

Not Applicable.

## Supplementary Materials

The following supporting information can be downloaded at: https://www.mdpi.com/article/10.3390/toxics11110899/s1, Table S1: Physicochemical analysis and concentration of heavy metals in wastewater; Table S2: Analysis of heavy metals using ANOVA in the wastewater and wastewater irrigation soil from three distinct areas of Kirri Shamozai, Dera Ismail Khan, KPK, Pakistan; Table S3: Physicochemical analysis and concentration of heavy metals mg/kg in wastewater irrigation soil of Kiri Shamozai, Dera Ismail Khan, KPK, Pakistan; Table S4: Heavy metals transfer factors for vegetables grown in Kirri Shamozai, Dera Ismail Khan, KPK, Pakistan; Table S5: DIM and HRI for adults and children consuming vegetables produced in zone-KS wastewater irrigation soil; Table S6: DIM and HRI for adults and children consuming vegetables produced in zone-FA wastewater irrigation soil; Table S7: DIM and HRI for adults and children consuming vegetables produced in zone-JM wastewater irrigation soil.
