# Supplementary material for: Statistical Analysis and Health Risk Assessment: Vegetables Irrigated with Wastewater in Kirri Shamozai, Pakistan
Source: Toxics. 2023 Nov 2;11(11):899. doi: 10.3390/toxics11110899 (PMC10674659; doi:10.3390/toxics11110899)
Supplement: Supplementary file 1 [file toxics-11-00899-s001.zip › toxics-2658160-supplementary-done.pdf]

Table S1. Physicochemical analysis and concentration of heavy metals in wastewater.

	Range	Minimum	Maximum	Mean	Std. Deviation	Skewness	Kurtosis	WHO (2007)
**Zone-KS (Wastewater)**								
pH value (H^+)	0.89	7.54	8.43	7.9700	0.36487	0.243	1.351	6.5-8.5
EC (**μS/cm**)	195	1695	1890	1769	91.37	0.923	0-.89	1400
TDS (mg/L)	189	1089	1278	1173	78.21	0.762	1.72	1000
Cu(mg/L)	0.12	0.28	0.40	0.36	0.06	-1.82	3.38	0.2
Fe(mg/L)	2.96	8.98	11.9	10.5	1.31	0.02	-2.39	5
Zn(mg/L)	0.25	0.10	0.34	0.26	0.11	-1.77	3.18	2
Mn(mg/L)	0.07	0.27	0.35	0.32	0.0	-1.38	1.35	0.2
Pb(mg/L)	0.27	0.20	0.47	0.34	0.12	0-.31	-2.66	0.1
Cd(mg/L)	0.03	0.57	0.60	0.58	0.02	0.51	-2.83	0.01
Ni(mg/L)	0.11	0.29	0.39	0.36	0.05	-1.91	3.70	0.2
Cr(mg/L)	0.28	0.59	0.86	0.73	0.13	-0.28	-3.18	0.01
**Zone-FA (Wastewater)**								
pH value (H^+)	0.91	7.48	8.39	7.78	0.41	1.74	3.09	6.5-8.5
EC (**μS/cm**)	174	1692	1866	175	79.73	1.58	2.35	1400
TDS (mg/L)	341	1089	1430	1202	159.8	1.46	1.71	1000
Cu(mg/L)	0.13	0.25	0.38	0.32	0.06	0.01	-0.04	0.2
Fe(mg/L)	2.01	7.83	9.84	8.78	0.82	0.67	0.38	5
Zn(mg/L)	0.18	0.20	0.38	0.26	0.07	0.01	1.62	2
Mn(mg/L)	0.08	0.29	0.37	0.31	0.04	0.01	1.97	0.2
Pb(mg/L)	0.16	0.20	0.35	0.26	0.08	0.007	0.04	0.1
Cd(mg/L)	0.12	0.48	0.59	0.53	0.05	0.003	0.18	0.01
Ni(mg/L)	0.31	0.27	0.59	0.37	0.14	0.02	1.65	0.2
Cr(mg/L)	0.17	0.72	0.88	0.83	0.08	0.006	-1.96	0.01
**Zone-JM (Wastewater)**								
pH value (H^+)	0.96	7.02	7.98	7.66	0.45	-1.45	1.70	6.5-8.5
EC (**μS/cm**)	210	1260	1470	1372	89.58	-.42	-0.41	1400
TDS (mg/L)	176	606	782	678	76.73	1.015	0.57	1000
Cu(mg/L)	0.19	0.15	.35	0.22	0.08	1.41	1.60	0.2
Fe(mg/L)	2.09	4.98	7.07	5.75	0.92	1.46	2.19	5
Zn(mg/L)	0.08	0.01	0.09	0.05	0.03	-0.29	-3.95	2
Mn(mg/L)	0.09	0.19	0.27	0.21	0.03	1.88	3.65	0.2
Pb(mg/L)	0.04	0.05	0.09	0.07	0.01	-1.30	1.23	0.1
Cd(mg/L)	0.02	0.01	0.03	0.01	0.01	1.99	3.96	0.01
Ni(mg/L)	0.12	0.07	0.20	0.15	0.05	-1.69	3.03	0.2
Cr(mg/L)	0.13	0.09	0.22	0.14	0.06	0.133	-5.22	0.01

Table S2. Analysis of heavy metals using ANOVA in the wastewater and wastewater irrigation soil from three distinct areas of Kirri Shamozai, Dera Ismail Khan, KPK, Pakistan.

Zones	Pb	Cr	Cd	Cu	Zn	Ni	Fe	Mn
**Wastewater**								
Zone-KS	0.239a	0.655a	0.389a	0.323a	0.243a	0.349a	8.547a	0.302a
Zone-FA	0.222a	0.593 ab	0.367a	0.303 a	0.205a	0.315a	8.487a	0.281a
Zone-JM	0.158a	0.463 b	0.350a	0.2967a	0.107a	0.247a	7.933a	0.270a
Significant	ns	*	ns	ns	ns	ns	ns	ns

**Wastewater Irrigated Soil**								
Zone-KS	72.93a	84.57a	2.60a	30.95a	30.10a	73.95a	391.2a	277.4a
Zone-FA	67.97ab	76.14a	2.33a	28.36ab	26.5ab	60.36 a	374.4a	260.4a
Zone-JM	67.42ab	61.3a	2.06 ab	22.86 b	22.65 b	58.6a	352.4b	243.5a
Significant	ns	ns	*	ns	ns	*	**	ns

Letters a, and b show significant differences, *stands for significant at 0.05, **for significant at 0.01, and ns for not significant.

Table S3. Physicochemical analysis and concentration of heavy metals mg/kg in wastewater irrigation soil of Kiri Shamozai, Dera Ismail Khan, KPK, Pakistan.

	Range	Minimum	Maximum	Mean	Std. Deviation	Skewness	Kurtosis	WHO (2007)
**Zone-KS (Wastewater Irrigated Soil)**								
pH value(H^+)	0.37	8.62	8.99	8.80	0.16	0.14	-1.40	-
EC (µS/cm)	105	458	563	510.7	46.1	-0.02	-2.06	-
OM	1.54	1.05	2.59	1.76	0.66	0.37	-1.12	-
Cu(mg/kg)	15.1	24.6	39.7	32.6	7.01	-0.23	-3.53	36
Fe(mg/kg)	40	429	469	451.0	21.0	-0.12	-5.29	150
Zn(mg/kg)	6.0	28.1	34.1	30.2	2.68	1.65	2.87	300
Mn(mg/kg)	110.6	289.1	399.7	337.4	52.7	0.38	-3.53	500
Pb(mg/kg)	26	68.0	94.0	81.0	12.4	0.00	-4.47	85
Cd(mg/kg)	1.40	1.70	3.10	2.65	0.64	-1.86	3.61	0.8
Ni(mg/kg)	35.1	60.8	95.9	73.5	15.5	1.55	2.62	67.9
Cr(mg/kg)	48.0	73.0	121.0	92.	21.5	1.03	0.14	100
**Zone-FA (Wastewater Irrigated Soil)**								
pH value (H^+)	0.36	8.29	8.65	8.46	0.17	0.02	-5.078	-
EC (µS/cm)	93	477	570	51	41.5	0.66	-1.34	-
OM	1.71	1.09	2.79	1.83	0.70	0.88	1.88	-
Cu(mg/kg)	10.8	26.1	36.9	33.0	4.77	-1.58	2.71	36
Fe(mg/kg)	57.0	422	479	445	24.25	1.23	2.03	150
Zn(mg/kg)	14.2	22.6	36.8	28.9	5.95	0.72	0.99	300
Mn(mg/kg)	80.3	270	350.5	304.2	36.5	0.65	-1.76	500
Pb(mg/kg)	18.0	68.0	86.0	77.2	8.22	-0.11	-3.27	85
Cd(mg/kg)	1.60	1.20	2.80	2.12	0.72	-0.67	-1.67	0.8
Ni(mg/kg)	23.2	56.1	79.3	66.85	9.55	0.52	1.48	67.9
Cr(mg/kg)	30.0	69.0	99.0	85.0	16.2	-0.05	-5.69	100
**Zone-JM (Wastewater Irrigated Soil)**								
pH value (H^+)	0.38	7.82	8.20	8.02	0.16	-0.42	0.14	-
EC (µS/cm)	142	308	450	378	65.5	.039	-3.61	-
OM	1.69	1.40	3.09	2.03	0.76	1.19	0.60	-
Cu(mg/kg)	3.96	17.9	21.8	19.5	1.68	1.34	2.53	36
Fe(mg/kg)	46.90	178.7	225.6	203.5	19.9	-0.38	-0.42	150
Zn(mg/kg)	6.47	16.4	22.9	19.4	2.67	0.65	1.44	300
Mn(mg/kg)	15.3	111.3	126.6	118.3	6.42	0.54	0.48	500
Pb(mg/kg)	15.3	32.03	47.3	39.9	6.34	-0.27	0.53	85
Cd(mg/kg)	0.50	1.40	1.90	1.57	0.22	1.72	3.26	0.8
Ni(mg/kg)	7.57	39.1	46.6	43.3	3.18	-.070	0.83	67.9
Cr(mg/kg)	2.43	33.5	35.9	34.6	1.0	0.43	0.99	100

Table S4. Heavy metals transfer factors for vegetables grown in Kirri Shamozai, Dera Ismail Khan, KPK, Pakistan.

	Zones	Cu	Fe	Zn	Mn	Pb	Cd	Ni	Cr
	KS	0.98	0.05	0.79	0.19	0.48	1.95	0.74	0.36
Spinacia oleracea	FA	0.96	0.04	0.79	0.20	0.48	3.14	0.75	0.42
	JM	0.87	0.26	0.98	0.23	0.20	1.33	0.78	1.11
	KS	0.78	0.07	0.63	0.04	0.56	3.47	0.57	0.44
Brassica oleracea var. capitala	FA	0.80	0.07	0.79	0.04	0.53	3.76	0.60	0.45
	JM	0.77	0.19	0.25	0.14	0.15	1.12	0.12	0.81
	KS	0.88	0.04	0.41	0.09	0.49	1.21	0.27	0.47
Brassica oleracea var. botrytis	FA	0.90	0.04	0.52	0.10	0.47	2.00	0.26	0.38
	JM	0.91	0.29	0.52	0.02	0.15	1.26	0.63	0.40
	KS	0.43	0.08	0.54	0.04	0.47	1.56	0.54	0.08
Raphanus sativus	FA	0.46	0.09	0.61	0.05	0.39	2.42	0.48	0.08
	JM	0.85	0.19	0.94	0.05	0.20	1.33	0.68	0.17
	KS	0.44	0.02	0.46	0.17	0.15	3.04	0.42	0.18
Brassica rapa subsp.	FA	0.52	0.03	0.59	0.20	0.18	4.14	0.33	0.19
	JM	0.54	0.05	0.46	0.15	0.17	0.49	0.41	0.35
	KS	0.02	0.68	0.11	0.21	1.73	0.70	0.16	0.52
Benincasa fistulosa	FA	0.56	0.02	0.73	0.11	0.23	2.38	0.71	0.18
	JM	0.73	0.04	0.86	0.25	0.23	0.56	0.65	0.26
	KS	0.68	0.07	0.34	0.11	0.23	3.78	0.42	0.31
Daucus carota subsp. Sativus	FA	0.83	0.07	0.33	0.12	0.56	3.57	0.41	0.32
	JM	0.55	0.03	0.44	0.07	0.15	1.19	0.61	0.49
	KS	0.46	0.06	0.62	0.12	0.20	3.34	0.99	0.33
Lactuca sativa	FA	0.46	0.06	0.69	0.13	0.23	4.23	0.97	0.31
	JM	0.72	0.14	0.95	0.12	0.23	1.19	0.58	0.70

Table S5. DIM and HRI for adults and children consuming vegetables produced in zone-KS wastewater irrigation soil.

Vegetables		Cu	Fe	Zn	Mn	Pb	Cd	Ni	Cr
Spinacia oleracea					Adult				
	DIM	1.63×10^{-2}	1.15×10^{-2}	1.20×10^{-2}	3.30×10^{-2}	1.99×10^{-2}	2.36×10^{-3}	2.57×10^{-2}	1.62×10^{-2}
	HRI	4.07×10^{-1}	1.65×10^{-2}	4.01×10^{-2}	1.00	4.97	2.36	1.29	1.08×10^{-2}
					Child				
	DIM	1.88×10^{-2}	1.33×10^{-2}	1.39×10^{-2}	3.81×10^{-2}	2.29×10^{-2}	2.7×10^{-3}	2.96×10^{-2}	1.87×10^{-2}
	HRI	4.69×10^{-1}	1.90×10^{-2}	4.62×10^{-2}	1.15	5.73	2.71	1.48	1.25×10^{-2}
Brassica oleracea					Adult				
	DIM	1.30×10^{-2}	1.68×10^{-2}	9.64×10^{-3}	7.28	2.30×10^{-2}	4.19×10^{-3}	2.00×10^{-2}	1.99×10^{-2}
	HRI	3.26×10^{-1}	2.39×10^{-2}	3.21×10^{-2}	2.21×10^{-1}	5.76	4.19	9.98×10^{-1}	1.33×10^{-2}
					Child				
	DIM	1.50×10^{-2}	1.93×10^{-2}	1.11×10^{-2}	8.38×10^{-3}	2.65×10^{-2}	4.82×10^{-3}	2.30×10^{-2}	2.29×10^{-2}
	HRI	3.75×10^{-1}	2.76×10^{-2}	3.70×10^{-2}	2.54×10^{-1}	6.63	4.82	1.15	1.53×10^{-2}

Brassica oleracea var. botrytis	Adult								
	DIM	1.47x10^{-2}	9.95 x10^{-3}	6.28 x10^{-3}	1.56 x10^{-2}	2.04 x10^{-2}	1.47 x10^{-3}	9.64 x10^{-3}	2.15 x10^{-2}
	HRI	3.67x10^{-1}	1.42 x10^{-2}	2.09 x10^{-2}	4.71 x10^{-1}	5.11 x10^{-3}	1.47	4.82 x10^{-1}	1.43 x10^{-2}
	Child								
	DIM	1.69x10^{-2}	1.15 x10^{-2}	7.24 x10^{-3}	1.79 x10^{-2}	2.35 x10^{-2}	1.69 x10^{-3}	1.11 x10^{-2}	2.47 x10^{-2}
	HRI	4.22x10^{-1}	1.64 x10^{-2}	2.41 x10^{-2}	5.43 x10^{-1}	5.88 x10^{-3}	1.69	5.55 x10^{-1}	1.65 x10^{-2}
Rapanus sativus	Adult								
	DIM	7.23x10^{-3}	1.99 x10^{-2}	8.33 x10^{-3}	8.22 x10^{-3}	1.94 x10^{-2}	1.89 x10^{-3}	1.90 x10^{-2}	3.67 x10^{-3}
	HRI	1.81x10^{-1}	2.84 x10^{-2}	2.78 x10^{-2}	2.49 x10^{-1}	4.84	1.89 x10^{-3}	9.50 x10^{-1}	2.44 x10^{-3}
	Child								
	DIM	8.32x10^{-3}	2.29 x10^{-2}	9.59 x10^{-3}	9.47 x10^{-3}	2.23 x10^{-2}	2.17 x10^{-3}	2.19 x10^{-2}	4.22 x10^{-3}
	HRI	2.08x10^{-1}	3.27 x10^{-2}	3.20 x10^{-2}	2.87 x10^{-1}	5.58	2.17	1.09	2.81 x10^{-3}
Brassica rapa. subsp	Adult								
	DIM	7.33x10^{-3}	6.28 x10^{-3}	7.07 x10^{-3}	3.07 x10^{-2}	6.28 x10^{-3}	3.67 x10^{-3}	1.48 x10^{-2}	8.38 x10^{-3}
	HRI	1.83x10^{-1}	8.98 x10^{-3}	2.36 x10^{-2}	9.31 x10^{-1}	1.57	3.67	7.41 x10^{-1}	5.59 x10^{-3}
	Child								
	DIM	8.44x10^{-3}	7.24 x10^{-3}	8.14 x10^{-3}	3.54 x10^{-2}	7.24 x10^{-3}	4.22 x10^{-3}	1.71 x10^{-2}	9.65 x10^{-3}
	HRI	2.11x10^{-1}	1.03 x10^{-2}	2.7 x10^{-2}	1.07	1.81	4.22	8.53 x10^{-1}	6.43 x10^{-3}
Benincasa fistulosa	Adult								
	DIM	7.33x10^{-3}	6.28 x10^{-3}	7.07 x10^{-3}	3.07 x10^{-2}	6.28 x10^{-3}	3.67 x10^{-3}	1.48 x10^{-2}	8.38 x10^{-3}
	HRI	1.83x10^{-1}	8.98 x10^{-3}	2.36 x10^{-2}	9.31 x10^{-1}	1.57	3.67	7.41 x10^{-1}	5.59 x10^{-3}
	Child								
	DIM	9.53x10^{-3}	5.43 x10^{-3}	1.19 x10^{-2}	2.30 x10^{-2}	1.03 x10^{-2}	2.41 x10^{-3}	2.79 x10^{-2}	8.44 x10^{-3}
	HRI	2.38x10^{-1}	7.75	3.98 x10^{-2}	6.96 x10^{-1}	2.56	2.41	1.40	9.33 x10^{-1}
Daucus carota. subsp	Adult								
	DIM	1.14x10^{-2}	1.73 x10^{-2}	5.18 x10^{-3}	2.05 x10^{-2}	9.43 x10^{-3}	4.56 x10^{-3}	1.48 x10^{-2}	1.41 x10^{-2}
	HRI	2.84x10^{-1}	2.47 x10^{-2}	1.73 x10^{-2}	6.22 x10^{-1}	2.36	4.56	7.41 x10^{-1}	9.43 x10^{-3}
	Child								
	DIM	1.31x10^{-2}	1.99 x10^{-2}	5.97 x10^{-3}	2.36 x10^{-2}	1.09 x10^{-2}	5.25 x10^{-3}	1.71 x10^{-2}	1.63 x10^{-2}
	HRI	3.27x10^{-1}	2.84 x10^{-2}	1.99 x10^{-2}	7.16 x10^{-1}	2.71	5.25	8.53 x10^{-1}	1.09 x10^{-2}
Lactuca sativa	Adult								

	DIM	8.38x10^{-3}	4.03 x10^{-3}	3.46 x10^{-2}	1.52 x10^{-2}	8.93 x10^{-3}	1.79 x10^{-2}	1.09 x10^{-2}	2.49 x10^{-2}
	HRI	2.09	4.03	1.73	1.01 x10^{-2}	2.23 x10^{-1}	2.56 x10^{-2}	3.62 x10^{-2}	7.55E-01
	Child								
	DIM	8.93x10^{-3}	1.79 x10^{-2}	1.09 x10^{-2}	2.49 x10^{-2}	9.65 x10^{-3}	4.64x10^{-3}	3.98 x10^{-2}	1.75 x10^{-2}
	HRI	2.23x10^{-1}	2.56 x10^{-2}	3.62 x10^{-2}	7.55 x10^{-1}	2.41	4.64	1.99	1.17 x10^{-2}

Table S6. DIM and HRI for adults and children consuming vegetables produced in zone-FA wastewater irrigation soil.

Vegetables		Cu	Fe	Zn	Mn	Pb	Cd	Ni	Cr
Spinacia oleracea	Adult								
	DIM	1.56 x10^{-2}	1.05 x10^{-2}	1.10 x10^{-2}	3.25 x10^{-2}	1.94 x10^{-2}	3.46 x10^{-3}	2.52 x10^{-2}	1.83 x10^{-2}
	HRI	3.89 x10-	1.50 x10^{-2}	3.68 x10^{-2}	9.85 x10^{-1}	4.84	3.4	1.2	1.2 x10^{-2}
	Child								
	DIM	1.79 x10^{-2}	1.21 x10^{-2}	1.27 x10^{-2}	3.74 x10^{-2}	2.23 x10^{-2}	3.98 x10^{-3}	2.90x10^{-2}	2.11 x10^{-2}
	HRI	4.48 x10^{-1}	1.72 x10^{-2}	4.24 x10^{-2}	1.13	5.5	3.9	1.4	1.41 x10^{-2}
Brassica oleracea	Adult								
	DIM	1.30 x10^{-2}	1.73 x10^{-2}	1.11 x10^{-2}	7.38	2.09 x10^{-2}	4.14 x10^{-3}	2.01 x10^{-2}	1.99 x10^{-2}
	HRI	3.26 x10^{-1}	2.47 x10^{-2}	3.70 x10^{-2}	2.24 x10^{-1}	5.24	4.14	1.00	1.33 x10^{-2}
	Child								
	DIM	1.50 x10^{-2}	1.99 x10^{-2}	1.2 x10^{-2}	8.50 x10^{-3}	2.41 x10^{-2}	4.76 x10^{-3}	2.31 x10^{-2}	2.29 x10^{-2}
	HRI	3.75 x10^{-1}	2.84 x10^{-2}	4.26 x10^{-2}	2.54 x10^{-1}	6.03	4.76	1.15	1.53 x10^{-2}
Brassica oleracea var. botrytis	Adult								
	DIM	1.45 x10^{-2}	9.95 x10^{-3}	7.33 x10^{-3}	1.69 x10^{-2}	1.89 x10^{-2}	2.20 x10^{-3}	8.69 x10^{-3}	1.6 x10^{-2}
	HRI	3.63 x10^{-1}	1.42 x10^{-2}	2.44 x10^{-2}	5.13 x10^{-1}	4.71	2.20	4.35x10^{-1}	1.43 x10^{-2}
	Child								
	DIM	1.67 x10^{-2}	1.15 x10^{-2}	8.44 x10^{-3}	1.95 x10^{-2}	2.17 x10^{-2}	2.53 x10^{-3}	1.00x10^{-2}	1.93 x10^{-2}
	HRI	4.18 x10^{-1}	1.64 x10^{-2}	2.81 x10^{-2}	5.90 x10^{-1}	5.43	2.53	5.01x10^{-1}	1.29 x10^{-2}
Rapanus sativus	Adult								
	DIM	7.44 x10^{-3}	2.25 x10^{-2}	8.54 x10^{-3}	8.43 x10^{-3}	1.57 x10^{-2}	2.67 x10^{-3}	1.62 x10^{-2}	3.67 x10^{-3}
	HRI	1.861x10^{-1}	3.22 x10^{-2}	2.85 x10^{-2}	2.55 x10^{-1}	3.93	2.67	8.12 x10^{-1}	2.44 x10^{-3}
	Child								
	DIM	8.56 x10^{-3}	2.59 x10^{-2}	9.83 x10^{-3}	9.71 x10^{-3}	1.81 x10^{-2}	3.08 x10^{-3}	1.87x10^{-2}	4.22 x10^{-3}

	HRI	2.14×10^{-1}	3.70×10^{-2}	3.28×10^{-2}	2.9×10^{-1}	4.52	3.08	9.3×10^{-1}	2.81×10^{-3}
Brassica rapa. subsp	Adult								
	DIM	8.43×10^{-3}	7.33×10^{-3}	8.38×10^{-3}	3.26×10^{-2}	7.33×10^{-3}	4.56×10^{-3}	1.10×10^{-2}	8.38×10^{-3}
	HRI	2.11×10^{-1}	1.05×10^{-2}	2.79×10^{-2}	9.87×10^{-1}	1.83	4.56	5.50×10^{-1}	5.59×10^{-3}
	Child								
	DIM	9.71×10^{-3}	8.44×10^{-3}	9.65×10^{-3}	3.75	8.44×10^{-3}	4.22×10^{-3}	1.27×10^{-2}	9.65×10^{-3}
	HRI	2.43×10^{-1}	1.21×10^{-2}	3.22×10^{-2}	1.14	2.11	5.25	8.53×10^{-1}	6.43×10^{-3}
Benincasa fistulosa	Adult								
	DIM	9.06×10^{-3}	4.92×10^{-3}	1.0×10^{-2}	1.8×10^{-2}	9.43×10^{-3}	2.62×10^{-3}	2.39	7.85×10^{-3}
	HRI	2.26×10^{-1}	7.03×10^{-3}	3.42×10^{-2}	5.67×10^{-1}	2.36	2.62	1.2	5.24×10^{-3}
	Child								
	DIM	1.04×10^{-2}	5.67×10^{-3}	1.18×10^{-2}	2.15×10^{-2}	1.09×10^{-2}	3.02×10^{-3}	2.76×10^{-2}	9.05×10^{-3}
	HRI	2.61E-01	8.10×10^{-3}	3.94×10^{-2}	6.52×10^{-1}	2.71	3.0	1.38	6.03×10^{-3}
Daucus carota. subsp	Adult								
	DIM	1.35×10^{-2}	1.78×10^{-2}	4.66×10^{-3}	1.95×10^{-2}	2.25×10^{-2}	3.93×10^{-3}	1.40×10^{-2}	1.41×10^{-2}
	HRI	3.38×10^{-1}	2.54×10^{-2}	1.55×10^{-2}	5.92×10^{-1}	5.63	3.93	6.99×10^{-1}	9.43×10^{-3}
	Child								
	DIM	1.56×10^{-2}	2.04×10^{-2}	5.37×10^{-3}	2.25×10^{-2}	2.59×10^{-2}	4.52×10^{-3}	1.61×10^{-2}	1.63×10^{-2}
	HRI	3.89×10^{-1}	2.9×10^{-2}	1.79×10^{-2}	6.82×10^{-1}	6.48	4.52	8.05×10^{-1}	1.09×10^{-2}
Lactuca sativa	Adult								
	DIM	7.49×10^{-3}	1.56×10^{-2}	9.74×10^{-3}	2.16×10^{-2}	9.43×10^{-3}	4.66×10^{-3}	3.26×10^{-2}	1.36×10^{-2}
	HRI	1.87×10^{-1}	2.22×10^{-2}	3.25×10^{-2}	6.55×10^{-1}	2.36	4.66	1.63	9.08×10^{-3}
	Child								
	DIM	8.62×10^{-3}	1.79×10^{-2}	1.12×10^{-2}	2.49×10^{-2}	1.09×10^{-2}	5.37×10^{-3}	3.75×10^{-2}	1.57×10^{-2}
	HRI	2.16×10^{-1}	2.56×10^{-2}	3.74×10^{-2}	7.55×10^{-1}	2.71	5.37	1.88	1.05×10^{-2}

Table S7. DIM and HRI for adults and children consuming vegetables produced in zone-JM wastewater irrigation soil.

**Vegetables**		**Cu**	**Fe**	**Zn**	**Mn**	**Pb**	**Cd**	**Ni**	**Cr**
Spinacia oleracea	Adult								
	DIM	8.12×10^{-3}	2.78×10^{-2}	9.7×10^{-3}	1.42×10^{-2}	4.19×10^{-3}	9.95×10^{-4}	1.73×10^{-2}	1.99×10^{-2}

	HRI	2.03 x10^{-1}	3.96 x10^{-2}	3.25 x10^{-2}	4.30 x10^{-1}	1.05	9.95 x10^{-1}	8.67 x10^{-1}	1.33 x10^{-2}
	Child								
	DIM	9.35 x10^{-3}	3.20 x10^{-2}	1.12 x10^{-2}	1.63 x10^{-2}	4.82 x10^{-3}	1.15 x10^{-3}	2.00 x10^{-2}	2.29 x10^{-2}
	HRI	2.34 x10^{-1}	4.57 x10^{-2}	3.74 x10^{-2}	4.95 x10^{-1}	1.21	1.15	9.98 x10^{-1}	1.53 x10^{-2}
Brassica oleracea	Adult								
	DIM	7.17 x10^{-3}	2.04 x10^{-2}	2.57 x10^{-3}	8.75 x10^{-3}	3.14 x10^{-3}	8.38 x10^{-4}	2.76 x10^{-3}	1.47 x10^{-2}
	HRI	1.79 x10^{-1}	2.92 x10^{-2}	8.55 x10^{-3}	2.65 x10^{-1}	7.85 x10^{-1}	8.38 x10^{-1}	1.3 x10^{-1}	9.78 x10^{-3}
	Child								
	DIM	8.26 x10^{-3}	2.35 x10^{-2}	2.95 x10^{-3}	1.01 x10^{-2}	3.62 x10^{-3}	9.65 x10^{-4}	3.18 x10^{-3}	1.69 x10^{-2}
	HRI	2.07 x10^{-1}	3.36 x10^{-2}	9.85 x10^{-3}	2.54 x10^{-1}	9.05 x10^{-1}	9.65 x10^{-1}	1.59 x10^{-1}	1.12 x10^{-2}
Brassica oleracea var. botrytis	Adult								
	DIM	8.48 x10^{-3}	3.09 x10^{-2}	5.18 x10^{-3}	1.52 x10^{-3}	3.14 x10^{-3}	9.43 x10^{-4}	1.40 x10^{-2}	7.33 x10^{-3}
	HRI	2.12	4.41 x10^{-2}	1.73 x10^{-2}	4.60 x10^{-2}	7.85 x10^{-1}	9.43 x10^{-1}	6.99 x10^{-1}	4.89 x10^{-3}
	Child								
	DIM	9.77 x10^{-3}	3.56 x10^{-2}	5.97 x10^{-3}	1.75 x10^{-3}	3.62 x10^{-3}	1.09 x10^{-3}	1.61 x10^{-2}	8.44 x10^{-3}
	HRI	2.44 x10^{-1}	5.08 x10^{-2}	1.99 x10^{-2}	5.30 x10^{-2}	9.05 x10^{-1}	1.0	8.05 x10^{-1}	5.63 x10^{-3}
Rapanus sativus	Adult								
	DIM	7.91 x10^{-3}	2.04 x10^{-2}	9.32 x10^{-3}	3.04 x10^{-3}	4.19 x10^{-3}	9.95 x10^{-4}	1.51 x10^{-2}	3.14 x10^{-3}
	HRI	1.98 x10^{-1}	2.92 x10^{-2}	3.11 x10^{-2}	9.20 x10^{-2}	1.05	9.95 x10^{-1}	7.57 x10^{-1}	2.09 x10^{-3}
	Child								
	DIM	9.11 x10^{-3}	2.35 x10^{-2}	1.07 x10^{-2}	3.50 x10^{-3}	4.82 x10^{-3}	1.15 x10^{-3}	1.87 x10^{-2}	3.62 x10^{-3}
	HRI	2.28 x10^{-1}	3.36 x10^{-2}	3.58 x10^{-2}	1.06 x10^{-1}	1.21	1.15	8.71 x10^{-1}	2.4 x10^{-3}
Brassica rapa. subsp	Adult								
	DIM	5.08 x10^{-3}	6.28 x10^{-3}	4.61 x10^{-3}	9.58 x10^{-3}	3.67 x10^{-3}	3.67 x10^{-4}	1.10 x10^{-2}	6.28 x10^{-3}
	HRI	1.27	8.98 x10^{-3}	1.54 x10^{-2}	2.90 x10^{-1}	9.16 x10^{-1}	3.67 x10^{-1}	4.63 x10^{-1}	4.19 x10^{-3}
	Child								
	DIM	5.85 x10^{-3}	7.24 x10^{-3}	5.31 x10^{-3}	1.10 x10^{-2}	4.22 x10^{-3}	4.22 x10^{-4}	1.0 x10^{-2}	7.24 x10^{-3}
	HRI	1.46 x10^{-1}	1.03 x10^{-2}	1.77 x10^{-2}	3.34 x10^{-1}	1.06	4.22 x10^{-1}	5.34 x10^{-1}	4.82 x10^{-3}
Benincasa fistulosa	Adult								
	DIM	6.86 x10^{-3}	4.71 x10^{-3}	8.54 x10^{-3}	1.51 x10^{-2}	4.71 x10^{-3}	4.19 x10^{-4}	1.46 x10^{-2}	4.71 x10^{-3}
	HRI	1.71 x10^{-1}	6.73 x10^{-3}	2.85 x10^{-2}	4.59 x10^{-1}	1.18	4.19 x10^{-1}	7.31 x10^{-1}	3.14 x10^{-3}
	Child								

	DIM	1.04 x10^{-2}	5.67 x10^{-3}	1.18 x10^{-2}	2.1 x10^{-2}	1.09 x10^{-2}	3.02 x10^{-3}	2.7 x10^{-2}	9.05 x10^{-3}
	HRI	2.61 x10^{-1}	8.10 x10^{-3}	3.94 x10^{-2}	6.52 x10^{-1}	2.71	3.02	1.38	6.03 x10^{-3}
Daucus carota. subsp	Adult								
	DIM	5.13 x10^{-3}	3.67 x10^{-3}	4.40 x10^{-3}	4.40 x10^{-3}	3.14 x10^{-3}	8.90 x10^{-4}	1.37 x10^{-2}	8.90 x10^{-3}
	HRI	1.28 x10^{-1}	5.24 x10^{-3}	1.47 x10^{-2}	1.33 x10^{-1}	7.85 x10^{-1}	8.90 x10^{-1}	6.86 x10^{-1}	5.93 x10^{-3}
	Child								
	DIM	5.91 x10^{-3}	4.22 x10^{-3}	5.07 x10^{-3}	5.07 x10^{-3}	3.62 x10^{-3}	1.03 x10^{-3}	1.58 x10^{-2}	1.0 x10^{-2}
	HRI	1.48 x10^{-1}	6.03 x10^{-3}	1.69 x10^{-2}	1.54 x10^{-1}	9.05 x10^{-1}	1.03	7.90 x10^{-1}	6.83 x10^{-3}
Lactuca sativa	Adult								
	DIM	6.70 x10^{-3}	1.52 x10^{-2}	9.48 x10^{-3}	7.44 x10^{-3}	4.71 x10^{-3}	8.90 x10^{-4}	1.29 x10^{-2}	1.26 x10^{-2}
	HRI	1.68 x10^{-1}	2.17 x10^{-2}	3.16 x10^{-2}	2.25 x10^{-1}	1.18	8.90 x10^{-1}	6.44 x10^{-1}	9.08 x10^{-3}
	Child								
	DIM	7.72 x10^{-3}	1.75 x10^{-2}	1.0 x10^{-2}	8.56 x10^{-3}	5.43 x10^{-3}	1.03 x10^{-3}	1.48 x10^{-2}	1.45 x10^{-2}
	HRI	1.93 x10^{-1}	2.50 x10^{-2}	3.64 x10^{-2}	2.59 x10^{-1}	1.36	1.03	7.42 x10^{-1}	4.95 x10^{-1}
